# *LetsTalkShots*: personalized vaccine risk communication

**DOI:** 10.3389/fpubh.2023.1195751

**Published:** 2023-06-30

**Authors:** Daniel A. Salmon, Matthew Z. Dudley, Janesse Brewer, Jana Shaw, Holly B. Schuh, Tina M. Proveaux, Amelia M. Jamison, Amanda Forr, Michelle Goryn, Robert F. Breiman, Walter A. Orenstein, Lee-Sien Kao, Robina Josiah Willock, Michelle Cantu, Tori Decea, Robin Mowson, Kate Tsubata, Lucie Marisa Bucci, Jaqueline Lawler, James D. Watkins, Jamie W. Moore, James H. Fugett, Adriele Fugal, Yazmine Tovar, Marie Gay, Aleen M. Cary, Iulia Vann, Lee B. Smith, Lilly Kan, Magda Mankel, Sumayya Beekun, Victoria Smith, Stephanie D. Adams, Steven A. Harvey, Peter Z. Orton

**Affiliations:** ^1^Institute for Vaccine Safety, Johns Hopkins University Bloomberg School of Public Health, Baltimore, MD, United States; ^2^Department of International Health, Johns Hopkins University Bloomberg School of Public Health, Baltimore, MD, United States; ^3^Department of Health, Behavior, and Society, Johns Hopkins University Bloomberg School of Public Health, Baltimore, MD, United States; ^4^Department of Public Health and Preventive Medicine, State University of New York, Upstate Medical University, Syracuse, NY, United States; ^5^Department of Pediatrics, State University of New York, Upstate Medical University, Syracuse, NY, United States; ^6^Department of Epidemiology, Johns Hopkins Bloomberg School of Public Health, Baltimore, MD, United States; ^7^Department of Global Health, Rollins School of Public Health, Emory University, Atlanta, GA, United States; ^8^Division of Infectious Diseases, Department of Medicine, Emory University School of Medicine, Atlanta, GA, United States; ^9^ieas42.org, New York, NY, United States; ^10^Department of Community Health and Preventive Medicine, Morehouse School of Medicine, Atlanta, GA, United States; ^11^Department of Immunization, National Association of County and City Health Officials, Washington, DC, United States; ^12^Bonnemaison, Baltimore, MD, United States; ^13^Bucci-Hepworth Health Services Inc., Pincourt, QC, Canada; ^14^Orange County Department of Health, Goshen, NY, United States; ^15^Williams County Combined Health District, Montpelier, OH, United States; ^16^Guilford County Division of Public Health, Greensboro, NC, United States; ^17^Monongalia County Health Department, Morgantown, WV, United States; ^18^Utah County Health Department, Provo, UT, United States; ^19^Border Studies Program, Earlham College, Tucson, AZ, United States; ^20^Center for Indigenous Health, Johns Hopkins University Bloomberg School of Public Health, Baltimore, MD, United States; ^21^Center for Global Health Innovation, Atlanta, GA, United States

**Keywords:** vaccine hesitancy, communication, COVID-19, vaccines, tailored application

## Abstract

**Introduction:**

Vaccine hesitancy is a global health threat undermining control of many vaccine-preventable diseases. Patient-level education has largely been ineffective in reducing vaccine concerns and increasing vaccine uptake. We built and evaluated a personalized vaccine risk communication website called *LetsTalkShots* in English, Spanish and French (Canadian) for vaccines across the lifespan. *LetsTalkShots* tailors lived experiences, credible sources and informational animations to disseminate the right message from the right messenger to the right person, applying a broad range of behavioral theories.

**Methods:**

We used mixed-methods research to test our animation and some aspects of credible sources and personal narratives. We conducted 67 discussion groups (*n* = 325 persons), stratified by race/ethnicity (African American, Hispanic, and White people) and population (e.g., parents, pregnant women, adolescents, younger adults, and older adults). Using a large Ipsos survey among English-speaking respondents (*n* = 2,272), we tested animations aligned with vaccine concerns and specific to population (e.g., parents of children, parents of adolescents, younger adults, older adults).

**Results:**

Discussion groups provided robust feedback specific to each animation as well as areas for improvements across animations. Most respondents indicated that the information presented was interesting (85.5%), clear (96.0%), helpful (87.0%), and trustworthy (82.2%).

**Discussion:**

Tailored vaccine risk communication can assist decision makers as they consider vaccination for themselves, their families, and their communities. *LetsTalkShots* presents a model for personalized communication in other areas of medicine and public health.

## Introduction

Vaccine hesitancy was declared a top-10 public health threat by the World Health Organization (WHO) just prior to the emergence of the SARS-CoV-2 virus in 2019. Europe was experiencing a measles resurgence, largely fueled by vaccine concerns (1). The United States (US) almost lost measles elimination status as there were reoccurring outbreaks in geographical (2), cultural (3), and religious (4) clusters of under-vaccinated populations. Measles can be the “canary in the coalmine” for the impact of vaccine refusal, as two doses of measles-containing vaccine are extremely effective, the disease is one of the most highly transmissible infections, and there are many opportunities for importation from areas of the world where measles is not well controlled (5). Vaccine hesitancy has also contributed to the resurgence of pertussis and poor control of HPV and influenza-associated illnesses in the US (6). Local health department immunization programs indicated reported vaccine hesitancy and vaccine confidence and immunization rates were a top priority (7). In July 2022, a case of vaccine-derived poliovirus was confirmed in an unvaccinated person from this same religious population which had previously experienced a measles outbreak. Given the asymptomatic nature of polio, and poliovirus being found in multiple sewage samples, there have likely been thousands of undetected cases.

Educational interventions about vaccines for patients (and/or their parents) have largely not been shown to positively impact vaccine decision-making and vaccine uptake (8), and can even backfire among the most vaccine hesitant persons (9). For example, educational efforts aimed at increasing vaccine uptake to control the spread of COVID-19 have been hampered by substantial proportions of the population questioning the need for and benefits of vaccination, along with a wide range of concerns including vaccines' speed of development and safety. Vaccine acceptance and concerns have varied by political affiliation (prioritization of personal liberties vs. community good), trust in public health authorities (government response), and many socio-demographic factors, such as race/ethnicity, age, education (10, 11). Vaccine equity concerns have also been prominent especially because of the disproportionate impact of disease in historically underserved and vulnerable populations.

As described by WHO, “messages need to be tailored for the specific target group, because messaging that too strongly advocates vaccination may be counterproductive, reinforcing the hesitancy of those already hesitant” (12). Personalized medicine aims to improve patient care by tailoring diagnoses, risk assessments, and therapy based on patient geographical and ethnic variability within populations (13). Similarly, personalized health communication has the potential to improve decision-making, and is consistent with medical and public health recommendations to tailor messages to individual patient interests (14–17). Our group has focused on addressing vaccine hesitancy and supporting vaccination decision-making, often resulting in increased vaccine acceptance, through an effective personalized communication tool that can widely disseminate tailored messaging from credible messengers at the individual level (18–20).

We started with *MomsTalkShots*, a website that tailors vaccine information to pregnant women, mothers, and their friends and families (to cocoon and protect the infant) (21). *MomsTalkShots* began with a short questionnaire that captured patient-level socio-demographic characteristics and vaccine attitudes, beliefs, concerns, and intentions, and then algorithmically tailored which educational videos each user received based on their responses. A pregnant woman who already intended to vaccinate received a short message reinforcing the value of vaccination. Those with concerns received racially/ethnically congruent introductions from obstetricians and pediatricians (most commonly cited as credible sources for vaccine information) to engender trust and display empathy without reinforcing any myths. For example, a woman worried about vaccine ingredients while pregnant would see an obstetrician saying “it's understandable that she would want to be careful with everything that goes in her body when pregnant” (which connects on a shared value), rather than saying “it's understandable that she would be concerned about vaccine ingredients” (which validates a myth, even if later addressed). Then, through engaging animation, specific concerns were addressed based on the best available evidence, before a pivot to the risks of disease and the effectiveness of vaccination. Lastly, the obstetrician or pediatrician made a strong personal recommendation to vaccinate, such as “I strongly encourage all of my patients and family to get vaccinated”. In addition to Tailoring Theory (22), MomsTalkShots was informed by and included the constructs of the Health Belief Model (18), Bandura's Social Cognitive Theory (19), Salience (20), Psychological Reactance (23), and the Theory of Normative Conduct.

We rigorously evaluated *MomsTalkShots* through a randomized controlled trial (RCT) among 2,092 pregnant women, recruited from 23 geographically and socio-demographically diverse obstetric offices in Georgia and Colorado. We examined: (1) self-reported usability of *MomsTalkShots*; (2) vaccine knowledge, attitudes, and beliefs; and (3) maternal vaccine uptake (chart confirmed) and uptake among randomly selected family and friends (self-reported). The majority of mothers reported *MomsTalkShots* was helpful (95%), trustworthy (94%), interesting (97%), and clear (99%), and this did not vary by demographics or birth parity (21). *MomsTalkShots* resulted in a two-thirds reduction of mothers reporting a need for additional information to make an informed decision about vaccines, indicating it filled an information gap. Among women who had no intention of or were unsure about receiving influenza vaccine during pregnancy, those who accessed *MomsTalkShots* were 61% more likely to receive the influenza vaccine than those who did not, for an absolute increase in 13% more women getting vaccinated (chart confirmed) (24). Women accessing *MomsTalkShots* were more than 5 times more likely to be confident in infant vaccine safety and about 75% less likely to have specific concerns about infant vaccine safety 1 year after the birth of their infant, compared to those who did not access *MomsTalkShots* (25). In addition, family and friends who were shared *MomsTalkShots* for the purposes of cocooning along with a small pharmacy-based financial incentive for vaccination were almost seven times more likely to receive influenza vaccine than those who received the financial incentive without *MomsTalkShots* (26).

To our knowledge, *MomsTalkShots* is the only direct patient education shown by an RCT to increase chart-confirmed vaccine uptake and sustainably improve vaccine confidence. *MomsTalkShots* demonstrated the potential for a meaningful impact for clinical and public health practice. Based upon this successful model, we built and evaluated an expanded personalized vaccine risk communication website called *LetsTalkShots* which includes three languages (English, Spanish and French Canadian) and contains information for all recommended vaccines across the lifespan including COVID-19, tailoring content to adolescents, adults, parents, and pregnant women.

## Methods

### Development approach

We developed content tailored to each of the following relevant populations of vaccine decision-makers, chosen mostly to reflect differences in recommended vaccines by age: parents of infants (<2 years of age), parents of children (3–10 years of age), parents of adolescents (11–17 years of age), adolescents themselves (11–17 years of age), younger adults (18–50 years of age), older adults (>50 years of age), and pregnant women. We began by thoroughly reviewing the literature for vaccine knowledge, attitudes, and concerns among each of the above-mentioned populations, and specific to each disease and vaccine among these populations. Based upon this literature review and our own mixed-methods research in this area, we identified 74 topics to be addressed through animation (Supplementary Table 1). We then used our multi-disciplinary team to develop brief messages on each topic. Our team included expertise in infectious diseases, immunology, vaccine effectiveness, vaccine safety, clinical practice (including discussing immunization issues with patients/parents), risk perception, communication, and decision-making. This team was responsible for getting the science right, including being transparent about uncertainty in science, and presenting it in a manner that would be understandable to a diversity of individuals. A script writer then refined these messages and transformed them into scripts for animation, including the use of visual depictions and metaphors. For example, recognizing that many people struggle to understand risks, particularly rare risks, we highlighted portions of crowds in football stadiums of different sizes to visually depict and compare the risks of disease and the risks and benefits of vaccination. We expanded our use of behavior theories to include Narrative Theory Anecdotes (27–30). Timing Inoculation Theory (31), and the Transtheoretical Model for Behavioral Change (32), as described in Table 1. We had an iterative process between the script writer and the scientists to ensure that script narrations were appropriate for animation, stayed true to the science, and included application of our behavioral theories. Animations were designed to align closely with their spoken narration, such that all visual elements, especially those metaphoric, were consistent with the science and would accurately support their intended meaning. Any issues wherein the animation, narration or visual images created viewer confusion or misunderstanding were later corrected after discussion group feedback.

**Table 1:** Application of behavioral theory in *LetsTalkShots*.

**Behavioral theory**	**Application**
Tailoring	•Get the right message to the right person from the right messenger •Specific concerns only asked of people who indicate not confident getting vaccinated •Tailoring to specific concerns also helps avoid *mere exposure*/familiarity effects (inadvertently spreading misinformation/normalizing specific concerns)
Narrative Theory Anecdotes (22–25)/personal stories	Using personal stories of a racially/ethnically congruent person telling their personal story of how COVID-19 impacted them (COVID-19 only)
Timing Inoculation Theory (26)	Provide a small dose of arguments “from the other side” the audience is likely to encounter, so that they are more able to resist the message when they are subsequently exposed
Health Belief Model (15)	•Animation including disease susceptibility and severity, vaccine effectiveness and safety, and cue to action •Using football and soccer stadiums to visualize risk of vaccines and diseases and benefits of vaccination •Use of metaphors for concepts such as variable vaccine effectiveness as an umbrella providing partial protection during a heavy rain
Transtheoretical Model for behavioral change (27)	Tailoring call to action to where the person is on the hesitancy spectrum.
Bandura's Social Cognitive Theory (5)	Emphasizes role of self-efficacy or individuals' belief in their capability to perform a behavior by emphasizing choice
Salience (6)	•Visualization and metaphors to make the science clearer/more salient •Congruency in personal stories and credible sources
Psychological reactance (7)	Animation and overall messages framed as information to help people make their own decisions (rather than pressure to get a vaccine)
Theory of normative conduct	Benefit animation, which all people receive, emphasizes community benefits and impact of most people getting vaccinated

### Message testing

We used mixed-methods research to test our animation and aspects of our credible sources and personal narratives. We conducted 67 discussion groups (*n* = 325), stratified by race/ethnicity (African American, Hispanic, and White people) and population (e.g., parents, pregnant women, adolescents, younger adults, and older adults). Sessions lasted 90–120 min and sought to understand what information participants found most helpful, where there were “turn offs” or moments where they stopped listening, how well they understood (or did not understand) various pieces of messaging, and areas for improvements. Discussion groups were recruited through Ipsos, Qualtrics, and Schlessinger panels, and screened to identify vaccine hesitant persons. Participants were consented and given a financial incentive for participation. Discussion group recruitment included screener questions to ensure that participants held at least one of the vaccine concerns being addressed by the animations assigned to their session. Discussion guides were used to solicit feedback and groups were led by experienced and trained facilitators with experience working in vaccine hesitancy. Notetakers captured themes with attention to potential differences among racial/ethnic groups and areas for improvement specific to each piece of animation and to our approach more broadly.

We then conducted an online national panel survey of US adults to test the animations. A representative sample was selected through the Ipsos KnowledgePanel (www.ipsos.com), a probability-based web panel with about 60,000 members initially recruited by mail. Of the 5,323 panel members were stratified by race/ethnicity and randomly selected and emailed an invitation to complete this survey between September 1-12, 2022, 2,787 consented to and participated in the survey (52% completion rate). Households without internet access were provided tablet computers and internet access. Hispanics were supplementally recruited through random digit dialing of area codes with concentrated Hispanic populations, and the survey was offered in English and Spanish. Black and Hispanic respondents were oversampled by 50%. Enrollment quotas were used to ensure the sample's socio-demographic distribution approximated that of the US.

The survey captured the sociodemographic characteristics of respondents (gender, race/ethnicity, age, education, region, and political affiliation) as well as influenza and COVID-19 vaccination status, intentions, and concerns. Parents were identified and asked additional questions about the ages of their children, the vaccination status of each child, and if they had concerns around childhood vaccination. The survey used scales to measure confidence in vaccines and trust in the Centers for Disease Control and Prevention (CDC) using a previously developed and validated scale (33) found to be strongly correlated with vaccine attitudes, intention and acceptance among adults (for influenza and COVID-19 vaccines), parents (for pediatric vaccines) (34), and healthcare providers (35). The two scales were dichotomized at the median creating a “high” and “low” for each construct.

Among English-speaking respondents (*n* = 2,272 final sample size), animations aligned with vaccine concerns and specific to population (e.g., parents of children, parents of adolescents, younger adults, older adults) were delivered to respondents. Respondents without vaccine concerns viewed an animation reviewing the benefits of vaccines for their population. Respondents with concerns watched an animation specific to one of their concerns followed by the benefits of vaccines animation for their population. After completion of the animation(s), respondents were asked if they found the animation(s): (1) interesting; (2) clear; (3) helpful; and (4) trustworthy. Response options were a 4-point Likert scale (strongly disagree, disagree, agree, strongly agree). Responses to the four questions were coded and summed then divided by the maximum possible score to create a video feedback scale from 0 to 100, with 0 as the score for those who strongly disagreed to all four questions (e.g., those who gave completely negative feedback) and 100 as the score for those who strongly agreed to all four questions (e.g., those who gave completely positive feedback). This scale was then dichotomized at 50 to create an indicator differentiating between mostly positive vs. mostly negative feedback. The frequency of positive feedback overall and to each of the four questions individually was stratified by sociodemographic characteristics, vaccine status and intentions, confidence in vaccine safety, and trust in CDC. *P*-values were estimated using the Pearson chi-squared proportion test at a significance level of α = 0.05.

## Results

Discussion groups provided robust feedback specific to each animation as well as areas for improvements across animations. As all participants had vaccine concerns, based upon recruitment, some respondents would initiate comments with “while I am not anti-vaccine” and subsequently provide feedback which was very critical of the need for vaccines or their value and safety. Often, we received conflicting advice about the same animation, such as “give me more information” and “make it shorter,” or “more data and statistics” and “be more relatable,” or “be more definitive” and “you are oversimplifying, and it is more complicated and nuanced”. We found parents were the most opinionated and were more likely to report they had done their own research compared with adults who were not parents. Older adults seemed the most open-minded and willing to consider information. Despite a focus on identifying areas where there were differences in saliency, risk perception and preferred risk communication approaches by race/ethnicity, none were identified.

The major themes that arose from these discussion groups of vaccine-hesitant participants included being critical of calls-to-action to vaccinate, the use of absolute adjectives such as “best” and “safest”, and mention of the impact of vaccine refusal on other people which was perceived as guilting or shaming. Changes were made across animations in response. For example, we created a second version of the animation on benefits of vaccines for each population. Persons without vaccine concerns receive a version of the benefits animation which includes a call to-action to be vaccinated and generally takes a presumptive approach to vaccination, whereas persons with vaccine concerns receive a version of the benefits animation in which language was softened and revised to emphasize choice in vaccine decision-making. Similarly, we softened the language and emphasized choice in animations that respond to specific vaccine concerns. We also revised our language to humanize very rare vaccine-adverse reactions.

Discussion groups frequently expressed frustrations about poorly explained changing recommendations for masks and other COVID-19 prevention measures, contributing to distrust of vaccine recommendations. In response, we created a short piece of animation for those with COVID-19 vaccine concerns that acknowledges the challenges of an emerging pandemic and the consequent uncertainties, resulting in the need for recommendations to evolve as new science becomes available. Adolescents' focus groups indicated a preference for a younger and more energetic narrator, so new narration was recorded for adolescent animations accordingly. Additionally, every animation was revised based on feedback we received from multiple groups specific to each piece of animation.

In the Ipsos survey, most respondents agreed that the information presented was interesting (85.5%), clear (96.0%), helpful (87.0%), and trustworthy (82.2%). Based on these four questions, 85.9% of respondents provided an overall positive assessment of the animations. The frequency of positive feedback overall and to each of the four questions individually stratified by sociodemographic characteristics, vaccine status and intentions, confidence in vaccine safety, and trust in CDC (Table 2, Supplementary Table 1). Herein we report positive feedback by subpopulations.

**Table 2:** Positive feedback and reporting animations interesting, clear, helpful and trustworthy, stratified by sociodemographic characteristics, vaccine confidence and acceptance, and trust in the Centers for Disease Control and Prevention (CDC).

		**% Positive Feedback**	**% Agree**			
			**Interesting**	**Clear**	**Helpful**	**Trustworthy**
All		86	86	96	87	82
Gender	Female	89^*^	87^*^	98^*^	90^*^	83
	Male	83^*^	82^*^	94^*^	84^*^	82
Parent Status	No Children <18	87^*^	86^*^	96	88^*^	84^*^
	At Least One Child <18	83^*^	82^*^	95	86^*^	77^*^
MSA Status	Non-Metro	78^*^	79^*^	92^*^	82^*^	72^*^
	Metro	87^*^	86^*^	96^*^	88^*^	84^*^
Race/Ethnicity	White, Non-Hispanic	82^*^	79^*^	96	83^*^	79^*^
	Black, Non-Hispanic	91^*^	92^*^	96	93^*^	87^*^
	Other, Non-Hispanic	90^*^	90^*^	97	89^*^	88^*^
	Hispanic	86^*^	86^*^	96	87^*^	82^*^
Household Income	>$50K	86	86	93^*^	87	79
	$50–75K	89	87	98^*^	90	84
	$75–100K	86	86	96^*^	87	84
	$100–150K	86	86	98^*^	86	82
	$150K+	86	82	97^*^	86	84
Education	No high school diploma or GED	80^*^	76^*^	90^*^	83	76^*^
	High school graduate	82^*^	83^*^	93^*^	86	78^*^
	Some college or Associate's degree	86^*^	86^*^	97^*^	87	82^*^
	Bachelor's degree	87^*^	86^*^	98^*^	87	83^*^
	Master's degree or higher	90^*^	86^*^	98^*^	90	88^*^
Age	18–29	81^*^	79^*^	94^*^	86^*^	78^*^
	30–44	80^*^	77^*^	93^*^	82^*^	76^*^
	45–59	87^*^	86^*^	97^*^	87^*^	83^*^
	60+	91^*^	90^*^	98^*^	91^*^	88^*^
Political Affiliation	Republican	74^*^	75^*^	94^*^	75^*^	66^*^
	Democrat	95^*^	92^*^	98^*^	96^*^	94^*^
	Independent/Other	83^*^	82^*^	95^*^	86^*^	80^*^
Region	Northeast	88	84	98	89	84
	Midwest	84	83	95	84	80
	South	86	86	95	88	83
	West	86	86	96	87	81
Vaccinated against Influenza	No	76^*^	77^*^	93^*^	79^*^	69^*^
	Yes	94^*^	90^*^	98^*^	93^*^	92^*^
Vaccinated against COVID (at least one dose)	No	54^*^	62^*^	89^*^	60^*^	36^*^
	Yes	92^*^	89^*^	98^*^	92^*^	90^*^
Confidence in Vaccine Safety	Not Confident	62^*^	67^*^	87^*^	67^*^	46^*^
	Confident	92^*^	89^*^	98^*^	92^*^	92^*^
Trust in CDC	Low Trust	76^*^	77^*^	93^*^	78^*^	68^*^
	High Trust	97^*^	93^*^	99^*^	96^*^	98^*^

^*^p <0.05.

Positive feedback was statistically significantly more frequent among: females vs. males (88.7% vs. 83.3%); adults aged 45–59 (86.8%) and 60 years and older (91.1%) vs. those aged 18–29 (81.1%) and 30–44 years (79.9%); Black (90.8%) and Hispanic (86%) persons vs. White people (82.5%); those with higher education degrees (90.4% among those with a master's degree or more) vs. lower (80.0% among no high school diploma); metro (86.9%) vs. non-metro (78.0); and political affiliation (73.8% among Republicans, 83.4% among Independents, and 95.1% among Democrats). Positive feedback was much less frequent for COVID-19 vaccine videos (56.8%) than for videos about other routine vaccines, such as those recommended for children (78.1%), adolescents (70.7%), younger adults (71.6%) and older adults (77.1%). Positive feedback was statistically significantly more frequent among: those who had vaccinated against influenza (93.7%) in the previous season vs. those who had not (76.1%), those who had received at least one dose of COVID-19 vaccine (91.9%) vs. those who had not (54.5%), those confident in vaccine safety (92.3%) vs. those wo were not (61.9%), and those with high trust in CDC (97.3%) vs. those with low trust in CDC (75.9%). Frequency of positive feedback did not vary by income or region.

## Discussion

We created a broad range of animations^1^ explaining the benefits of, and responding to common concerns about, vaccines across the lifespan, and through a mixed-methods evaluation found them extremely well received across populations. There were clear differences by sociodemographic characteristics, trust in public health, vaccine hesitancy, and prior vaccine behavior. However, the animations were still well received even among subpopulations with lower reported usability. For example, 46% of persons who were not confident in the safety of vaccines and 68% of people with low trust in public health authorities reported the animation trustworthy—a difficult construct to achieve among the most vaccine-hesitant groups.

These animations are not intended to be used in isolation but rather are meant to be introduced and ended by racially/ethnically congruent credible speakers. For COVID-19, we also include videos of racially/ethnically congruent persons describing their lived experiences with COVID-19. We found that receiving a lived experience video prior to the informational animation increased the likelihood of listening to the entire animation by 9-fold, and that racial congruence between the credible source and user doubled the likelihood of viewing the entire animation (36).

Understanding that people are often most interested in hearing from people and credible sources from their own community, we locally tailored *LetsTalkShots* to 16 communities. We identified about 60 local credible sources in these communities, ranging from healthcare providers to the Governor of West Virginia to religious leaders. We made multiple versions of these final comments of credible sources so we could tailor the call-to-action to the hesitancy of the user. We also recorded about 60 lived experiences about COVID-19 from persons within these communities, ranging from pregnant women to parents of young children to family members. By entering their zip code, viewers from these 16 communities are shown credible sources and lived experiences from their own communities.

**Figure 1:**
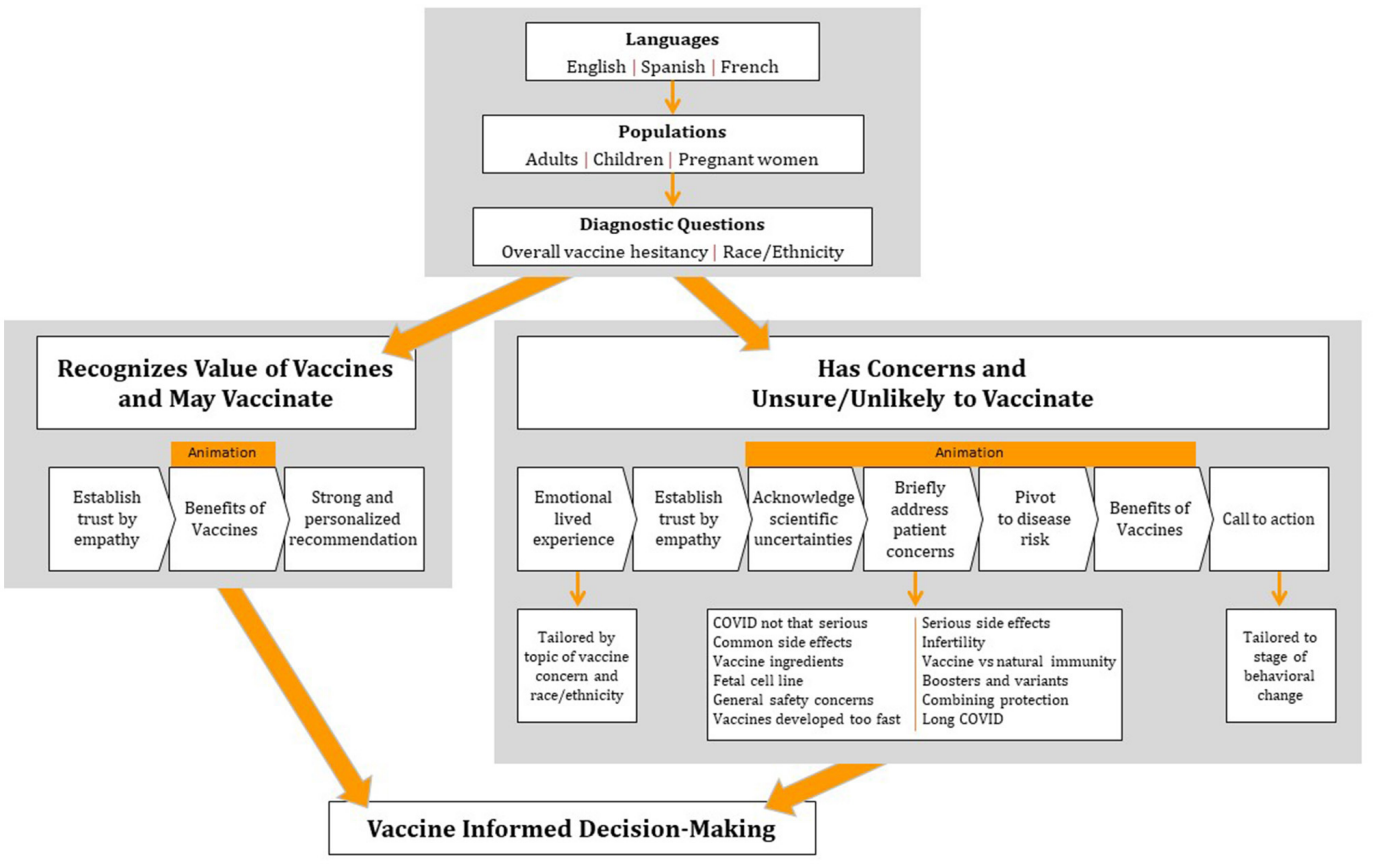
Tailoring algorithm for COVID-19.

In all, we developed approximately 10 h of video content (74 pieces of 2–4-min animations, 60 credible sources and 60 lived experiences). However, our platform is programmed so that each user receives only 3–8 min of content upfront. Which videos each user is shown is based on a short questionnaire given upfront that captures population, race/ethnicity, zip code, perceptions of disease risk, and vaccine intentions and concerns. Those without vaccine concerns receive a short introductory message from a racially/ethnically congruent credible source, one animation about the benefits of vaccination, and final comments from the credible source strongly recommending vaccination. Those with vaccine concerns first receive a racially/ethnically congruent lived experience (for COVID-19), an introduction from a racially/ethnically congruent credible source, an animation addressing one of their main concerns then pivoting to the risks of disease and benefits of vaccines, and then final comments from the credible source emphasizing choice and talking with their doctor. After viewing these videos, users are then provided a personalized video gallery with additional lived experience videos and animations addressing any other concerns they raised. An example of this algorithm for COVID-19 vaccines is depicted in Figure 1.

While not a panacea for the global challenge of vaccine hesitancy, tailored vaccine risk communication can assist decision makers as they consider vaccination for themselves, their families, and their communities. However, as COVID-19 has repeatedly demonstrated, and the public astutely noticed, the science and practice around disease and vaccines are often changing. For example, when the Novavax COVID-19 vaccine became available, we needed to revise several pieces of animation, which we were able to accomplish in 2 weeks and prior to the CDC recommendation for use. After testing our animation, a well conducted study was published finding a dose-response relationship between alum adjuvanted vaccines and persistent asthma (37). While this single study does not demonstrate a causal relationship, to remain scientifically accurate, we needed to quickly revise our vaccine ingredient animation for each age group. This situation highlights the need for keeping education materials up to date with new science and highlights that objective and credible vaccine communication is based on science. The most well-crafted messages cannot overcome gaps in science and the credibility of those who conduct the science. Communication strategies are just one arrow in the quiver of addressing hesitancy – particularly when hesitancy is grounded in historical trauma, institutional racism, and lack of access to services, as is the case in many vulnerable populations. Furthermore, science, no matter how well communicated, is only one factor in decision-making, which is a complex and individual calculation of information, values, the current context in which someone is making a decision, and access to services.

Scale-up is not an obstacle for *LetsTalkShots* from a technical standpoint. However, building this tool does not ensure it will be used. We are in the process of integrating *LetsTalkShots* into clinical practices so that the practice shares *LetsTalkShots* with their patients in advance of appointments. After the patient uses *LetsTalkShots*, their provider receives a profile of their patient's vaccine intentions and concerns, with specific talking points to help address each concern. We also include a provider training on how to talk to patients about vaccines and an electronically updated book providing clinicians information on vaccine-preventable diseases, vaccine recommendations, and the science around vaccine safety concerns (38). Evaluation of this multi-level, integrated approach to using *LetsTalkShots* for uptake of vaccines in populations beyond just pregnant women and new mothers would be beneficial. We are also partnering with public health and immunization partners and exploring social media strategies for directing general and specific audiences to the *LetsTalkShots* website.

*LetsTalkShots* can be tailored for other countries as we have done for Canada. However, the vaccine hesitancy issues in Canada and the US are similar, making this adaptation fairly straightforward, with added languages (French and Canadian accent for narration) and small differences in vaccine recommendations. Considerable formative and survey data would be required for adaptation to other countries, given potential differences in culture, language, values, and vaccine concerns.

## Limitations

Our theory-driven mixed-methods approach to personalized vaccine risk communication has limitations. Testing was conducted only in English, whereas we currently have Spanish and French versions available. Additional testing in populations that speak these languages would be helpful. While we largely reached saturation in discussion groups, there may have been differences within or between subpopulations that we did not identify. While our discussion groups were demographically diverse and held concerns that were addressed through animation, participants may not have been representative of the general population. Even though our survey was large and tested many pieces of animation using standardized usability outcomes, we were inadequately powered to identify small differences in usability between animations and doing so would require an extraordinarily large study. While Ipsos panels are nationally representative and have provided similar estimates for COVID-19 vaccine coverage as other sources such as the CDC (11), there is still the potential for selection bias, though the sampling strategy and use of demographic stratum should reduce this potential bias.

## Conclusions

*LetsTalkShots* provides a scalable tool for personalized vaccine risk communication. Many other areas of medicine and public health would benefit from similar personalized communication tools. Designing, evaluating and delivering the right message from the right messenger to the right person requires understanding the knowledge, attitudes, and behaviors in the target populations, and how these vary by subpopulations. Additionally, capturing personal stories and credible sources as well as using animation, while having many strengths, is an expensive endeavor. Compared to other areas of medicine and public health, vaccine recommendations are among the more complex and unfortunately controversial, perhaps making other areas for personalized risk communication more easily attainable. Personalizing risk communication has the potential to aid patient decision-making and improve uptake of medical and public health recommendations by tailoring messages to individual patient needs.

## Data availability statement

The raw data supporting the conclusions of this article will be made available by the authors, without undue reservation.

## Ethics statement

The studies involving human participants were reviewed and approved by Johns Hopkins Institutional Review Board. Written informed consent for participation was not required for this study in accordance with the national legislation and the institutional requirements.

## Author contributions

DS, JB, and LK contributed to the conception and design. DS and MD contributed to the analysis and interpretation of the data. DS, MD, JB, JS, HS, TP, AJ, AF, MG, RB, WO, L-SK, SA, VS, MC, RJ, SH, PO, SB, YT, LB, and IV contributed to drafting and revision. All authors have approved the submitted version.

## Footnotes

^1^ https://www.youtube.com/@instituteforvaccinesafety6495/videos
