# Corrigendum: *LetsTalkShots*: personalized vaccine risk communication

**DOI:** 10.3389/fpubh.2023.1311055

**Published:** 2023-10-30

**Authors:** Daniel A. Salmon, Matthew Z. Dudley, Janesse Brewer, Jana Shaw, Holly B. Schuh, Tina M. Proveaux, Amelia M. Jamison, Amanda Forr, Michelle Goryn, Robert F. Breiman, Walter A. Orenstein, Lee-Sien Kao, Robina Josiah Willock, Michelle Cantu, Tori Decea, Robin Mowson, Kate Tsubata, Lucie Marisa Bucci, Jaqueline Lawler, James D. Watkins, Jamie W. Moore, James H. Fugett, Adriele Fugal, Yazmine Tovar, Marie Gay, Aleen M. Cary, Iulia Vann, Lee B. Smith, Lilly Kan, Magda Mankel, Sumayya Beekun, Victoria Smith, Stephanie D. Adams, Steven A. Harvey, Peter Z. Orton

**Affiliations:** ^1^Institute for Vaccine Safety, Johns Hopkins University Bloomberg School of Public Health, Baltimore, MD, United States; ^2^Department of International Health, Johns Hopkins University Bloomberg School of Public Health, Baltimore, MD, United States; ^3^Department of Health, Behavior, and Society, Johns Hopkins University Bloomberg School of Public Health, Baltimore, MD, United States; ^4^Department of Public Health and Preventive Medicine, State University of New York, Upstate Medical University, Syracuse, NY, United States; ^5^Department of Pediatrics, State University of New York, Upstate Medical University, Syracuse, NY, United States; ^6^Department of Epidemiology, Johns Hopkins Bloomberg School of Public Health, Baltimore, MD, United States; ^7^Department of Global Health, Rollins School of Public Health, Emory University, Atlanta, GA, United States; ^8^Division of Infectious Diseases, Department of Medicine, Emory University School of Medicine, Atlanta, GA, United States; ^9^ieas42.org, New York, NY, United States; ^10^Department of Community Health and Preventive Medicine, Morehouse School of Medicine, Atlanta, GA, United States; ^11^Department of Immunization, National Association of County and City Health Officials, Washington, DC, United States; ^12^Bonnemaison, Baltimore, MD, United States; ^13^Bucci-Hepworth Health Services Inc., Pincourt, QC, Canada; ^14^Orange County Department of Health, Goshen, NY, United States; ^15^Williams County Combined Health District, Montpelier, OH, United States; ^16^Guilford County Division of Public Health, Greensboro, NC, United States; ^17^Monongalia County Health Department, Morgantown, WV, United States; ^18^Utah County Health Department, Provo, UT, United States; ^19^Border Studies Program, Earlham College, Tucson, AZ, United States; ^20^Center for Indigenous Health, Johns Hopkins University Bloomberg School of Public Health, Baltimore, MD, United States; ^21^Center for Global Health Innovation, Atlanta, GA, United States

**Keywords:** vaccine hesitancy, communication, COVID-19, vaccines, tailored application

In the published article, an author name was incorrectly written as Robina Josiah Willcock. The correct spelling is Robina Josiah Willock.

The authors apologize for this error and state that this does not change the scientific conclusions of the article in any way. The original article has been updated.

