# Supplementary material for: LetsTalkShots: personalized vaccine risk communication
Source: Front Public Health. 2023 Jun 30;11:1195751. doi: 10.3389/fpubh.2023.1195751 (PMC10348877; doi:10.3389/fpubh.2023.1195751)
Supplement: Supplementary file 1 [file Table_1.DOCX]

#### Supplemental Table 1: Animation Titles

| **ADOLESCENTS** | **ALL AGES - Misinformation** |
| --- | --- |
| - **How can I talk with my parents or caregiver about vaccines?** - **Is the HPV vaccine new?** - **What about my fertility later in life?** - **What are known side effects of vaccines recommended for me?** - **What ingredients are in vaccines recommended for me?** - **What should I know about my recommended vaccine schedule?** - **What should I know about the vaccines recommended for me?** | - **Are vaccines still needed in countries with clean water and good sanitation?** - **Immunity from vaccination vs. immunity from infection: What’s the difference?** - **Should I worry that a vaccine causes the disease it’s meant to prevent?** - **What's the deal with Thimerosal?** - **Which flu shot should I get if I’m allergic to eggs?** - **Why can’t I get the flu from the flu vaccine?** |
| **PREGNANT WOMAN** | **COVID** |
| - **What are known side effects of vaccines recommended for me?** - **What ingredients are in vaccines?** - **What should I know about possible pregnancy complications?** - **What should I know about the vaccines recommended for me while I’m pregnant?** | - **How does combining protections keep me and my family safe?** - **How is COVID like a soccer match?** - **Immunity from vaccination vs. immunity from infection: What’s the difference?** - **Variants and Boosters – what’s it all mean?** - **What about Long COVID?** - **Changing Advice** - **Vaccine Safety** - **COVID's Other Problems** - **Fetal Cell Line Questions** - **Serious Side FX** - **Temporary Vaccine Reactions** - **The Infertility Question** - **Vaccination and Pregnancy** - **Vaccine Benefits for Adults** - **Vaccine Development Speed** - **Vaccine for Children** - **Vaccine Ingredients** |
| **PARENTS OF BABIES** |  |
| - **How can I make sure I can afford vaccines for my baby?** - **What are the known side effects of vaccines for my baby?** - **What ingredients are in vaccines recommended for my baby?** - **What should I know about the vaccine schedule for my baby?** - **What should I know about vaccines for my baby?** |  |
| **PARENTS OF CHILDREN** |  |
| - **How can I make sure I can afford vaccines for my child?** - **What are known side effects of vaccines for my child?** - **What do we know about vaccines and autism?** - **What ingredients are in vaccines recommended for my child?** - **What should I know about the vaccine schedule for my child?** - **What should I know about the vaccines for my child?** |  |
| **PARENTS OF ADOLESCENTS** | **OTHER** |
| - **How can I make sure I can afford vaccines for my pre-teen or teen?** - **Is the HPV vaccine new?** - **What about my teen’s fertility later in life?** - **What are known side effects of vaccines recommended for my teen?** - **What ingredients are in vaccines recommended for my teen?** - **What should I know about the vaccine schedule for my teen?** - **What should I know about the vaccines recommended for my teen?** - **Will the HPV vaccine encourage my teen to be sexually active?** | - **How are vaccines like sports?** - **How can we be sure a vaccine is very safe?** - **How do vaccines work?** - **What are the different kinds of vaccines?** - **What if I have a problem with needles?** - **What if my child has a problem with needles?** - **What if my teen has a problem with needles?** - **What’s the Christian view of vaccines?** - **What’s the Islamic view of vaccines?** - **What’s the Jewish view of vaccines?** - **Why is the HPV vaccine important for me?** - **Why is vaccinating like prepping for a concert?** - **Why vaccinate children against rare diseases?** |
| **ADULT (<50)** |  |
| - **How can I make sure I can afford vaccines?** - **What are known side effects of vaccines recommended for me?** - **What ingredients are in vaccines recommended for me?** - **What should I know about the vaccines recommended for me?** |  |
| **OLDER ADULT (>50)** |  |
| - **How can I make sure I can afford vaccines?** - **What are known side effects of vaccines recommended for me?** - **What ingredients are in vaccines recommended for me?** - **What should I know about the vaccines recommended for me?** |  |
