# Supplementary material for: LetsTalkShots: personalized vaccine risk communication
Source: Front Public Health. 2023 Jun 30;11:1195751. doi: 10.3389/fpubh.2023.1195751 (PMC10348877; doi:10.3389/fpubh.2023.1195751)
Supplement: Supplementary file 2 [file Table_2.docx]

#### Supplemental Table 2: Odds of Positive Feedback and Reporting Animations Interesting, Clear, Helpful and Trustworthy, by Sociodemographic Characteristics, Vaccine Confidence and Acceptance, and Trust in the Centers for Disease Control and Prevention (CDC)

|  | | **Positive Feedback** | | **Interesting** | | **Clear** | | **Helpful** | | **Trustworthy** | |
| --- | --- | --- | --- | --- | --- | --- | --- | --- | --- | --- | --- |
|  | | **OR**  **(95%CI)*** | **p-value** | **OR**  **(95%CI)*** | **p-value** | **OR**  **(95%CI)*** | **p-value** | **OR**  **(95%CI)*** | **p-value** | **OR**  **(95%CI)*** | **p-value** |
| Gender | Female | ref |  | ref |  | ref |  | ref |  | ref |  |
|  | Male | **0.64**  **(0.50-0.81)** | **<0.01** | **0.66**  **(0.52-0.83)** | **<0.01** | **0.42**  **(0.26-0.67)** | **<0.01** | **0.59**  **(0.45-0.75)** | **<0.01** | 0.95  (0.77-1.18) | 0.66 |
| Parent Status | No Children <18 | ref |  | ref |  | ref |  | ref |  | ref |  |
|  | At Least One Child <18 | **0.70**  **(0.54-0.90)** | **0.01** | 0.78  (0.61-1.00) | 0.05 | 0.69  (0.44-1.07) | 0.10 | **0.75**  **(0.58-0.98)** | **0.03** | **0.63**  **(0.50-0.80)** | **<0.01** |
| MSA Status | Non-Metro | ref |  | ref |  | ref |  | ref |  | ref |  |
|  | Metro | **1.87**  **(1.35-2.59)** | **<0.01** | **1.52**  **(1.09-2.11)** | **0.01** | **2.22**  **(1.31-3.75)** | **<0.01** | **1.60**  **(1.13-2.26)** | **0.01** | **2.00**  **(1.48-2.70)** | **<0.01** |
| Race/Ethnicity | White, Non-Hispanic | ref |  | ref |  | ref |  | ref |  | ref |  |
|  | Black, Non-Hispanic | **2.09**  **(1.52-2.89)** | **<0.01** | **2.86**  **(2.06-3.98)** | **<0.01** | 1.06  (0.63-1.76) | 0.83 | **2.72**  **(1.90-3.87)** | **<0.01** | **1.77**  **(1.33-2.35)** | **<0.01** |
|  | Other, Non-Hispanic | 1.84  (0.99-3.42) | 0.05 | **2.27**  **(1.22-4.21)** | **0.01** | 1.25  (0.44-3.55) | 0.67 | 1.63  (0.89-2.97) | 0.11 | **1.91**  **(1.07-3.41)** | **0.03** |
|  | Hispanic | 1.30  (0.97-1.74) | 0.08 | **1.51**  **(1.13-2.00)** | **<0.01** | 1.15  (0.67-1.98) | 0.61 | **1.42**  **(1.05-1.92)** | **0.02** | 1.17  (0.90-1.53) | 0.24 |
| Household Income | Less than $50K | ref |  | ref |  | ref |  | ref |  | ref |  |
|  | $50-75K | 1.39  (0.96-2.03) | 0.08 | 1.25  (0.87-1.80) | 0.22 | **3.59**  **(1.68-7.69)** | **<0.01** | 1.36  (0.91-2.03) | 0.13 | **1.40**  **(1.01-1.95)** | **0.04** |
|  | $75-100K | 1.12  (0.76-1.65) | 0.58 | 1.01  (0.69-1.47) | 0.98 | **1.97**  **(1.01-3.86)** | **0.05** | 1.01  (0.67-1.51) | 0.97 | 1.39  (0.97-1.99) | 0.08 |
|  | $100-150K | 1.06  (0.74-1.50) | 0.76 | 1.00  (0.71-1.42) | 0.98 | **3.56**  **(1.66-7.63)** | **<0.01** | 0.83  (0.58-1.18) | 0.30 | 1.24  (0.90-1.71) | 0.20 |
|  | $150K+ | 1.06  (0.76-1.47) | 0.74 | 0.83  (0.61-1.13) | 0.24 | **2.08**  **(1.18-3.67)** | **0.01** | 0.94  (0.67-1.32) | 0.72 | 1.34  (0.99-1.82) | 0.06 |
| Education | No high school diploma or GED | ref |  | ref |  | ref |  | ref |  | ref |  |
|  | High school graduate | 1.14  (0.70-1.87) | 0.60 | 1.54  (0.96-2.47) | 0.08 | 1.46  (0.75-2.84) | 0.27 | 1.15  (0.68-1.96) | 0.60 | 1.15  (0.72-1.83) | 0.55 |
|  | Some college or Associate's degree | 1.57  (0.97-2.57) | 0.07 | **1.91**  **(1.20-3.04)** | **0.01** | **3.32**  **(1.63-6.74)** | **<0.01** | 1.41  (0.84-2.38) | 0.19 | 1.50  (0.95-2.36) | 0.08 |
|  | Bachelor's degree | 1.66  (1.00-2.76) | 0.05 | **1.77**  **(1.10-2.84)** | **0.02** | **6.07**  **(2.60-14.19)** | **<0.01** | 1.39  (0.81-2.37) | 0.23 | 1.57  (0.98-2.51) | 0.06 |
|  | Master’s degree or higher | **2.34**  **(1.35-4.05)** | **<0.01** | **2.00**  **(1.22-3.30)** | **0.01** | **5.69**  **(2.30-14.06)** | **<0.01** | **1.87**  **(1.05-3.31)** | **0.03** | **2.43**  **(1.46-4.05)** | **<0.01** |
| Age | 18-29 | ref |  | ref |  | ref |  | ref |  | ref |  |
|  | 30-44 | 0.92  (0.64-1.33) | 0.66 | 0.91  (0.64-1.28) | 0.58 | 0.85  (0.46-1.55) | 0.59 | 0.74  (0.50-1.10) | 0.14 | 0.86  (0.61-1.21) | 0.38 |
|  | 45-59 | **1.53**  **(1.04-2.25)** | **0.03** | **1.66**  **(1.14-2.42)** | **0.01** | 1.79  (0.90-3.54) | 0.09 | 1.11  (0.73-1.68) | 0.63 | 1.39  (0.97-1.99) | 0.08 |
|  | 60+ | **2.38**  **(1.62-3.49)** | **<0.01** | **2.49**  **(1.72-3.61)** | **<0.01** | **3.09**  **(1.52-6.26)** | **<0.01** | **1.65**  **(1.09-2.50)** | **0.02** | **1.96**  **(1.37-2.79)** | **<0.01** |
| Political Affiliation | Republican | ref |  | ref |  | ref |  | ref |  | ref |  |
|  | Democrat | **6.87**  **(4.75-9.93)** | **<0.01** | **4.10**  **(2.96-5.68)** | **<0.01** | **2.77**  **(1.50-5.10)** | **<0.01** | **7.26**  **(4.95-10.65)** | **<0.01** | **8.11**  **(5.79-11.36)** | **<0.01** |
|  | Independent/Other | **1.78**  **(1.36-2.33)** | **<0.01** | **1.52**  **(1.16-1.98)** | **<0.01** | 1.06  (0.65-1.74) | 0.82 | **1.95**  **(1.47-2.57)** | **<0.01** | **2.05**  **(1.60-2.64)** | **<0.01** |
| Region | Northeast | ref |  | ref |  | ref |  | ref |  | ref |  |
|  | Midwest | 0.71  (0.48-1.06) | 0.09 | 0.95  (0.66-1.38) | 0.80 | **0.43**  **(0.19-0.99)** | **0.05** | 0.69  (0.46-1.04) | 0.08 | 0.77  (0.54-1.10) | 0.15 |
|  | South | 0.84  (0.58-1.20) | 0.33 | 1.14  (0.82-1.58) | 0.44 | **0.42**  **(0.20-0.91)** | **0.03** | 0.90  (0.62-1.30) | 0.56 | 0.97  (0.70-1.34) | 0.85 |
|  | West | 0.77  (0.52-1.14) | 0.20 | 1.06  (0.74-1.52) | 0.74 | 0.53  (0.23-1.23) | 0.14 | 0.85  (0.57-1.27) | 0.42 | 0.81  (0.57-1.14) | 0.22 |
| Vaccinated against Influenza | No | ref |  | ref |  | ref |  | ref |  | ref |  |
|  | Yes | **4.65**  **(3.55-6.10)** | **<0.01** | **2.66**  **(2.09-3.38)** | **<0.01** | **4.15**  **(2.55-6.77)** | **<0.01** | **3.62**  **(2.77-4.74)** | **<0.01** | **5.24**  **(4.08-6.72)** | **<0.01** |
| Vaccinated against COVID (at least one dose) | No | ref |  | ref |  | ref |  | ref |  | ref |  |
|  | Yes | **9.44**  **(7.11-12.54)** | **<0.01** | **5.07**  **(3.85-6.69)** | **<0.01** | **4.87**  **(3.05-7.79)** | **<0.01** | **7.84**  **(5.88-10.45)** | **<0.01** | **16.43**  **(12.35-21.86)** | **<0.01** |
| Confidence in Vaccine Safety | Not Confident | ref |  | ref |  | ref |  | ref |  | ref |  |
|  | Confident | **7.41**  **(5.73-9.57)** | **<0.01** | **4.15**  **(3.25-5.30)** | **<0.01** | **9.45**  **(5.97-14.96)** | **<0.01** | **5.96**  **(4.59-7.74)** | **<0.01** | **13.15**  **(10.24-16.88)** | **<0.01** |
| Trust in CDC | Low Trust | ref |  | ref |  | ref |  | ref |  | ref |  |
|  | High Trust | **10.47**  **(7.92-13.83)** | **<0.01** | **5.20**  **(4.08-6.63)** | **<0.01** | **5.22**  **(3.31-8.2)** | **<0.01** | **8.44**  **(6.39-11.14)** | **<0.01** | **14.06**  **(10.81-18.29)** | **<0.01** |

*OR (95%CI) = Odds Ratio (95% Confidence Interval), ref = reference value, bold indicates statistical significance (p-value <0.05)
